# Research on Linear Actuators for Active Foil Bearings

**DOI:** 10.3390/ma15165694

**Published:** 2022-08-18

**Authors:** Łukasz Breńkacz, Rafał Kędra, Waldemar Janicki, Artur Maurin, Paweł Bagiński, Artur Andrearczyk, Beata Zima

**Affiliations:** 1 Institute of Fluid Flow Machinery, Polish Academy of Sciences, Fiszera 14, 80-231 Gdańsk, Poland; 2 Independent Researcher, 80-171 Gdańsk, Poland; 3 Institute of Ocean Engineering and Ship Technology, Gdansk University of Technology, G. Narutowicza 11/12, 80-233 Gdańsk, Poland

**Keywords:** active foil bearing, linear actuators, piezoelectric, SMA, stepper motor

## Abstract

Active foil bearings are a kind of gas foil bearing. They contain actuators which allow for modification of the bearing sleeve size and the shape of the lubrication gap. Rotor vibrations can be actively controlled by these changes. It is possible, among other things, to reduce the starting torque, control the vibration amplitude at different speeds and improve operational safety. Prototypes of active foil bearings are being developed based on different mechanical concepts. This paper provides an analysis of the linear mechanisms that are the base, and they are crucial for such developments. In the literature, there is a lack of characteristics of these actuators tested under real operating conditions of an active foil bearing. This article aims to fill this gap. New test rigs have been developed and used to precisely investigate the possibility of using actuators in active foil bearings. Since their geometry and control methods differ, the measurement systems have been adapted accordingly. The actuators studied were piezoelectrics, shape-memory alloys (SMAs) and stepper motors. Each of them was characterized by different operating characteristics. The results obtained allow for a comparison of the actuators. This approach is especially useful for the design and analysis of active foil bearings.

## 1. Introduction

The analysis of active foil bearings should begin with the traditional gas foil bearings, which have been developed for many years in numerous research centres around the world. Their design required a lot of both experimental and theoretical investigations. In 1970, Barnett and Silver first demonstrated that foil bearings can be widely used in high-speed turbomachines [1]. The development of foil bearing technology was presented in 2019 by Samanta et al. [2]. The authors pointed out that foil bearing technology is very promising as there are already many geometric solutions for foil bearings, which can be efficiently applied.

Kulkarni and Jana [3] presented the effect of geometric parameters of corrugated foil on the performance of foil bearings. The effect of the geometrical parameters on the load-carrying capacity of a multi-leaf gas bearing was presented by Li et al. [4]. The analysis of a structural model including gaps and friction was presented by Arghir and Benchekroun [5]. This paper presents the structural model of a bump-type foil bearing based on contact mechanisms. The model considers the elasticity of the top foil and the possibility of three types of gaps: between the rotor and the top foil, between the top foil and the bump foil and between the bump foil and the bearing sleeve. The model has been verified by experimental studies.

Foil bearings are characterized by an adaptive structure, as shown by Zywica and Bagiński [6]. Due to this foil structure, variability of the bearing sleeve diameter allows for adapting the foils of the active foil bearing to the current operating conditions.

Development of the foil bearings requires the use of advanced mathematical models and precisely tuned parameters, as well as extensive research to explain various aspects of their operation. Numerical and experimental studies on the temperature control of gas bearings were presented by Martowicz et al. [7]. A year later, Zdziebko and Martowicz extended these investigations, also showing the stress distribution within the bearing [8].

Because actuators are the main part of some mechatronic systems, it is always necessary to know their parameters to assess their influence on whole machines. In 2022, Xuan and Seung [9] wrote an article on state-of-the-art smart material actuators that have been created over the last decade, in which much attention was paid to their control aspects for various applications. This is a comprehensive review of smart material actuators, which focuses on their control aspects for various applications. The analysed actuators were actively applied to various control systems.

Sone et al. [10] presented a study on vibration suppression of cantilever beams using piezoelectric materials. The authors wrote that the piezoelectric vibration suppression technology utilises the inverse piezoelectric effect of piezoelectric materials to suppress the vibration of mechanisms through stress or strain. Chen et al. [11] presented a study on the experimental characterization of hysteresis in piezoceramic actuators. The authors showed the equipment used in their experimental study. The study was conducted with different excitations. The results consist of temperature and hysteresis loops of the actuator. Riccio et al. [12] presented the development of a combined micro-macro mechanics and used an analytical approach to design spring-based actuators with a shape-memory alloy, followed by experimental validation.

Tamburrano et al. [13] showed the design of servo valves driven by piezoelectric actuators. The authors wrote that the use of commercially available piezoelectric actuators such as piezo stacks amplified piezo stacks, rectangular benders and ring benders is very promising for the actuation of the main and pilot stages of servo valves. These actuators can also be used to develop a new design, and their characteristics are thoroughly discussed.

Romano and Tannuri [14] presented the modelling, control and experimental validation of a novel actuator based on shape-memory alloys. The paper presents the development of a mechanical actuator using a shape-memory alloy with a cooling system based on the thermoelastic effect. Experiments were conducted to evaluate the closed-loop performance, stability and robustness properties of the actuator. The results showed that the proposed cooling system and controller can improve the dynamic response of the actuator. Frequency characteristics were measured to verify dynamic performance. Xing et al. [15] presented a design and experiment of a new type of noncontact linear piezoelectric actuator modulated by an electromagnetic field. The proposed actuator uses electromagnetic force to modulate and transfer the locomotion between the stator and the runner. The drive scheme reduces the wear and friction between the stator and the runner.

Heya and Hirata [16] present an experimental verification of a three-degree-of-freedom electromagnetic actuator for image stabilization. This paper aims to develop a system to suppress the vibration. The principle of working here is different than the standard gimbal mechanism, which is widely used. The authors show the proposed actuator and its experimental verification.

In 2017, an example of preload control in a foil bearing was presented by Feng et al. [17]. The authors showed an active bump-type foil bearing (ABFB) in which preload can be controlled by changing the voltage applied to the piezoelectric actuators. The aim was to create a lubrication gap with variable geometry to improve bearing performance. The authors demonstrated that effective preload control is possible. In 2020, Guan et al. [18] presented a more extensive study of the same bearing. Their objective was to determine the rotordynamic characteristics of the rigid shaft supported on the ABFB. The authors showed the preload variations of the traditional foil bearing with hinges and piezoelectric actuators.

The tests were carried out to develop an active foil bearing. As there are many possible implementations, various design solutions were considered. It is possible to control the diameter of the foil bearing using the levers, as shown by Feng et al. [17].

When selecting the best possible actuators for the active foil bearing, many different types of actuators were considered. This paper summarizes the results of the laboratory tests performed on the three most promising types of actuators for this bearing. Moreover, these actuators were tested under real operating conditions, which is the main original element of the paper. Usually, the study presented in the literature is not devoted to actuators tested in real operational conditions. A review of active bearings, in which their mode of operation was taken into account, was presented by Breńkacz et al. [19]. There are many types of active bearings (including active foil bearings) in which the studied actuators are used or may be used in the future. An example may be the active gas bearing presented by Horikawa et al. [20]. Performance characteristics that have been obtained under similar temperature and load conditions to those of the active bearings can be directly used in the design process of these bearings. In general, the entire system (bearing) can be condensed over the selected degree of freedom (x) and considered as a one-dimensional dynamic problem.

**Figure 1:**
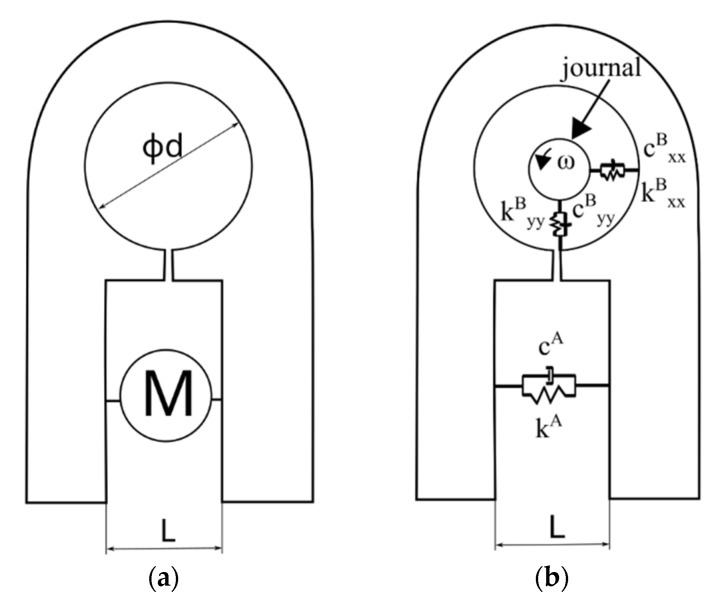
(**a**) Schematic of the active foil bearing; (**b**) Examples of discretization of degrees of freedom (in the direction of x and y respectively we obtain stiffness and damping parameters—k^B^xx, k^B^yy, c^B^xx, c^B^yy) and parameters of actuators (in the direction of the actuator, we obtain parameters like k^A^, c^A^, speed of changes, acceleration of changes).

If the chosen degree of freedom coincides with the motion direction of the actuator those two elements can be combined into a system of equations:

$$\left[\begin{array}{cc} m_{B} & 0\\ 0 & m_{A} \end{array}\right]\ddot{X}+\left[\begin{array}{cc} c_{B}+c_{A} & -c_{A}\\ -c_{A} & c_{A} \end{array}\right]\dot{X}+\left[\begin{array}{cc} k_{B}+k_{A} & -k_{A}\\ -k_{A} & k_{A} \end{array}\right]X=P\left(t\right)$$

where the index A is related to the actuator, the vector of displacements has form $X={\left\{\begin{array}{cc} x_{A} & x_{B} \end{array}\right\}}^{T}$ and P(t) is the force applied by the actuator. Based on the knowledge of the bearing and actuator parameters, it is possible to model the dynamics of the entire system. If necessary, an additional element describing other significant degrees of freedom, e.g., in the direction of the bearing diameter, can be introduced into the system of equations. During this work, several variants of the active foil bearing were considered. One of the variants was made using a 3D printer. Its shape is shown in Figure 1a. Dependences between the bearing parameters (two main of themes are stiffness and damping coefficients—k^B^_xx_, k^B^_yy_, c^B^_xx_, c^B^_yy_) and parameters of actuators are shown in Figure 1b.

**Figure 2:**
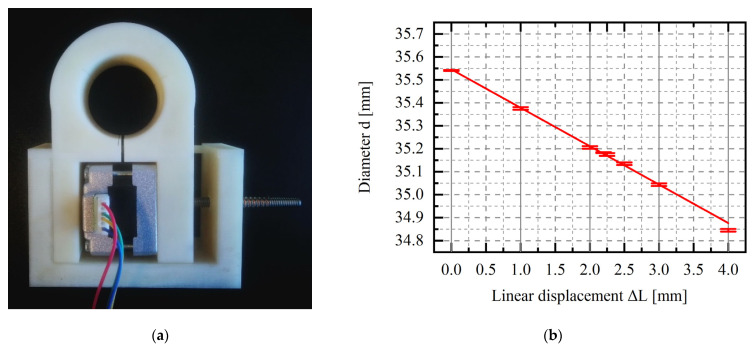
(**a**) The prototype of the active bearing was made using 3D printing (**b**) and sample results of preliminary research: diameter versus linear displacement of the stepper motor (designated by M); the initial value of L = 35.14 mm.

After the first test, in which the bearing diameter was changed, the bearing diameter was measured with a three-contact diameter gauge. The measured diameter of the bearing as a function of motor displacement is shown in Figure 2.

Linear displacement, which results in a change in bearing diameter, can be generated using various types of smart materials and stepper motors. Due to the applicable nature of the research conducted, the main selection criterion was the possibility of direct application of a ready-made component in the bearing, which means choosing a ready-made solution in the form of actuators. Three types of linear actuators, based on a piezoelectric element, a shape-memory alloy and a stepper motor, were selected from the range of available products.

In recent years, piezoelectric actuators have experienced significant development. The latest solutions combine broad working strokes with high precision and resolution. Moreover, piezoelectric actuators provide fast response, high stiffness, and actuation force [21]. There is a very large group of piezoelectric materials. Indeed, about a thousand types of piezoelectric crystals have been discovered, but only a few have found practical applications. Synthetic ceramics are most often used in linear actuators [22], although recently piezoelectric polymers have become more common [23]. Piezoelectric transducers made of synthetic ceramic materials are usually cuboid or cylinder-shaped, and to increase the operating range they are often stacked or integrated into amplifying devices such as levers or bridges [24].

Shape memory alloys (SMAs) are another group of smart materials used in the construction of linear actuators. These materials change shape when they are heated above a certain temperature. The physical phenomenon that is responsible for this is martensitic transformation. The elongation that occurs during this process can reach up to 8%. For this reason, SMA materials are typically used in actuators in the form of strands or thin wires. So far, SMA actuators have been widely used mainly in robotics [25] but they have also been successfully used in civil engineering [26].

A stepper motor is an electrical device powered by impulse voltage that causes a precisely defined rotor movement. The rotation is a multiple of a specific step, which corresponds to a certain angle of rotation [27]. Stepper motors have several important advantages, such as high accuracy, repeatability, ability to operate under high load or torque, and high durability. Due to these advantages, they are widely used in many industries. A wide variety of stepper motors, which are available on the market, find applications in many industries, such as the medical industry [28], robotics [29], and the mechanical industry.

For analysing rotating machinery, we measure among others the displacement of rotor shafts. It is easy to measure the trajectory of the rotating shaft, moment of torque, or acceleration of bearing supports. In the case of active bearings, there are a couple of new essential parameters. The most important in our opinion is the time of response, that is, the time that it takes to change from one diameter of bearing to the other one. The time, spread, and acceleration of this movement directly affect the movement of the rotating shaft. Essential parameters are also the operating range and the characteristics of actuators’ work parameters under loads and high temperature conditions. We can better describe the bearing and its working characteristics by knowing the characteristics of the whole mechanical system. All these parameters depend very much on the actuator characteristics.

The article presents the research results obtained for three selected elements: a piezoelectric stack, an SMA actuator, and a stepper motor tested in operational conditions. The main goal of the research presented in this paper was to determine the characteristics of the control signal (i.e., displacement) and to evaluate actuators in terms of use as active elements in the foil bearing.

## 2. Materials and Methods

The tests were conducted using a specially designed measurement system. The main assumption was to allow for the testing of various types of actuators with different ranges of motion, regardless of whether an actuator is linear or rotary.

**Figure 3:**
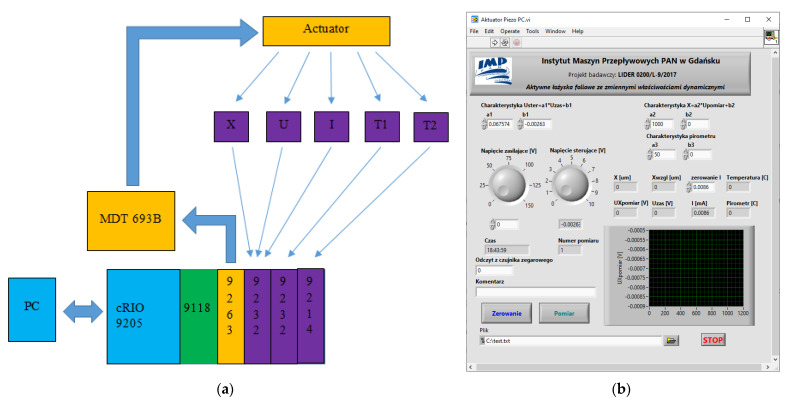
The measurement system used for the testing of piezoelectric actuators: (**a**) block diagram showing the hardware configuration; (**b**) the graphical user interface of the control application.

The measurement and control system was built based on the National Instruments’ CompactRIO automation controller. Depending on the type of actuator tested, it was supplemented with additional components. Figure 3a shows an example of a block diagram of the measurement and control system used to examine a piezoelectric element. The user communicates with the system via a personal computer (PC) and the authorial software transmits the desired voltage settings to the system controller (cRIO 9205), reads the measured parameters and records the data. The system controller and field-programmable gate array chassis(cRIO 9118) supervise the operation of the control module (NI-9263) and measurement modules (NI-9232 and NI-9214). The NI-9263 module generates a control voltage for the piezoelectric driver which provides a regulated source to power the piezoelectric actuator. The control is carried out in an open circuit. NI-9232 modules are responsible for measuring the signals coming from the sensors, i.e., the position of the mobile base of the actuator under test (X). To control the measurement system, a dedicated application has been created. The graphical user interface of the application is shown in Figure 3b.

The test rig was built using readily available mechanical components. Its main components are a vice and a lift, which is made of two bars and a steel plate. These elements are connected using linear bearings, which allow the plate to move in the vertical direction and ensure that the entire load is transferred to the actuator under test. Depending on the type of actuator, it can be freely placed on the vice or mounted in the steel jaws. The weight of the steel plate is approximately 1 kg. Due to its use, the actuator can be loaded during the test by placing additional weights. The actuator is controlled by a time-varying signal. The displacements were measured directly using a laser sensor KEYENC LK-H050 or indirectly using an encoder.

## 3. Experimental Analysis

### 3.1. Piezoelectric Actuator

The SA050536 piezoelectric stack actuator (made by PiezoDrive) was used for laboratory testing. The actuator has dimensions of 5 mm × 5 mm × 36 mm and was made in multilayer technology. The producer declares that with a supply voltage between −30 V and +150 V, the actuator can increase its length by 56 μm (0.156% strain) or generate an axial force of 900 N. The actuator has been factory-coated with a layer of polymer to protect it from environmental conditions. In addition, it is possible to equip it with spherical ends to reduce the risk of failure due to transverse loads. A model with a one-sided spherical end was used in the research.

#### 3.1.1. Research Program

**Figure 4:**
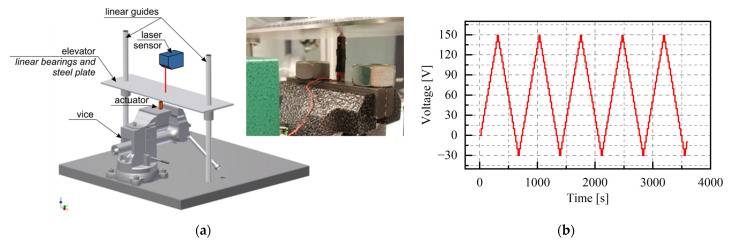
Experimental set-up used in piezoelectric research: (**a**) photo of the tested actuator placed between the vice and the measuring lift; (**b**) control signal voltage versus time.

The analysis of the piezoelectric actuator has been divided into two parts: static tests and dynamic tests. The piezoelectric actuator mounted on the test rig is shown in Figure 4a. The purpose of the first stage of the analysis (static tests) was to determine the actual variability of the actuator elongation (based on the data declared by the producer) and to analyse the influence of the initial load on the elongation range of the element. A research program has been developed based on preliminary measurements. The test started at zero volts and the voltage control signal was gradually increased by 10 volts during the 20 s until it reached a maximum value of +150 V. Then, the voltage was reduced to the minimum value (−30 V) with the same voltage step. The tests were terminated at zero voltage. The duration of each voltage level was chosen to obtain a stable value of the displacement; this was a quasi-static analysis. The stepped voltage as a function of time, obtained for five voltage cycles, is shown in Figure 4b.

The second dynamic part of the analysis was carried out using sinusoidal excitations with frequencies in the range of 1–50 Hz, for three signal amplitude ranges: 0–50 V, 0–100 V, 0–150 V and three preload levels (9.81 N, 49,05 N, and 98,1 N). In total, sixty-three different variants were considered. These tests, combined with the quasi-static analysis (which only makes it possible to determine the stiffness of the actuator), make it possible to determine a set of dynamic parameters and hysteresis parameters for example, for the Bouc-Wen or other phenomenological model. In both cases (static and dynamic analysis) the actuator was loaded by placing weights on the steel plate (the element of the measuring lift). The actuator was placed between the vice and the plate. The measurement was carried out by registering the displacements of the upper surface of the steel plate, at a point directly above the piezoelectric actuator spherical head.

#### 3.1.2. Results

**Figure 5:**
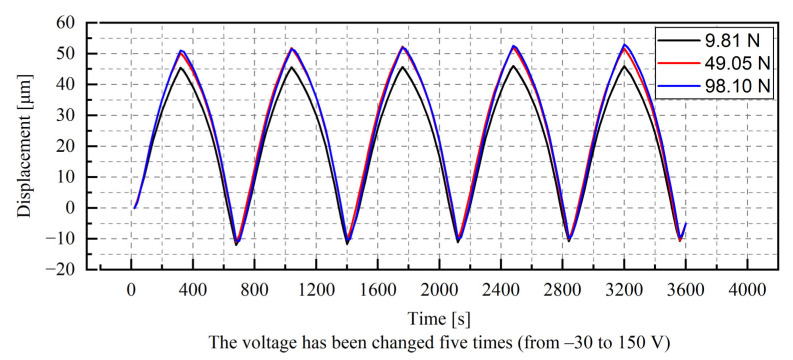
Results of the quasi-static test for different preload levels.

The exemplary results from the quasi-static analysis are shown in Figure 5. According to the data provided by the manufacturer, the displacement range of the stack is from about −10 μm to about 50 μm. For all the tests performed, the nonlinearity of the relationship between the voltage and the displacement is visible. It can be observed that the increase in displacements was slightly reduced with increasing voltage.

#### 3.1.3. Dynamic Properties

**Figure 6:**
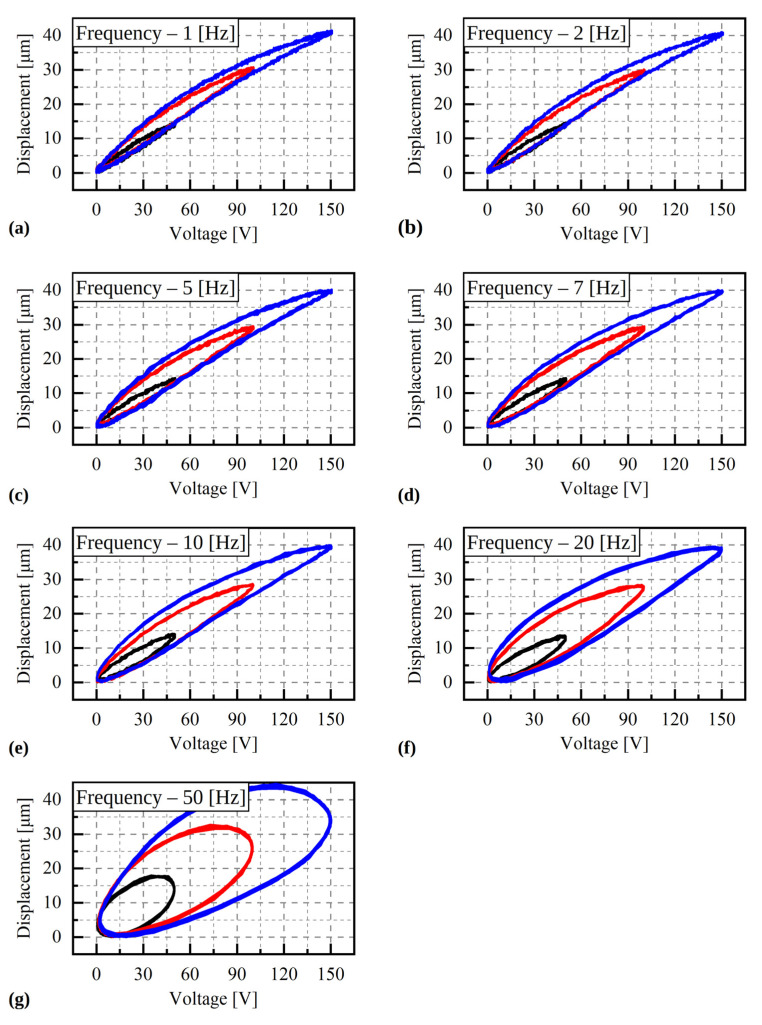
Results of the dynamic test for different excitation frequencies. (**a**)—1 Hz, (**b**)—2 Hz, (**c**)—5 Hz, (**d**)—7 Hz, (**e**)—10 Hz, (**f**)—20 Hz, (**g**)—50 Hz.

The results of the piezoelectric dynamic tests are shown in Figure 6. Each plot illustrates the displacements recorded for a number (at least 10) of sinusoidal voltage loops. The reproducibility of the results is much better than in the quasi-static tests. The loops obtained for frequencies between 1 and 10 Hz have similar shapes and for frequencies higher than 20 Hz, the vertical expansion of the loops is visible. Despite the different displacement patterns obtained for all analysed cases, similar displacement values were measured at the end of the cycle, namely 14 µm, 30 µm, and 40 µm, for the voltage ranges of 0–50 V, 0–100 V and 0–150 V, respectively. In addition, the maximum displacement value obtained for the nominal voltage (150 V) in the dynamic tests is significantly lower than the corresponding value measured in the quasi-static tests, due to inertia.

### 3.2. SMA Actuator

**Figure 7:**
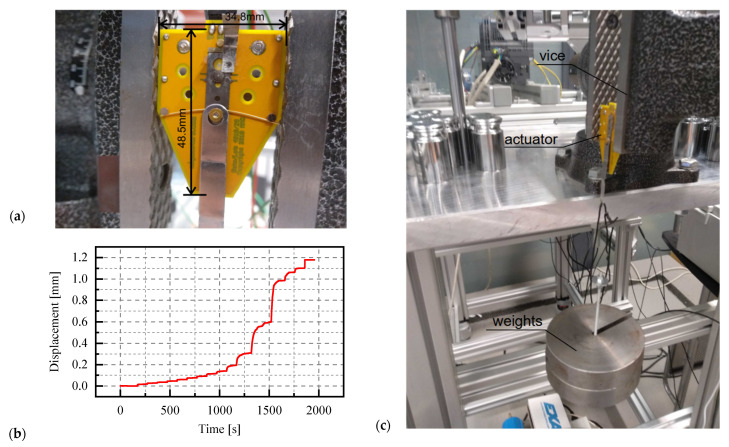
Experimental investigation of SMA actuator: (**a**) dimensions of the Miga iNITIator-062-010 actuator; (**b**) example of a graph showing displacement versus time; (**c**) Actuator during measurement.

The Miga iNITIator-062-01`0 actuator, manufactured by Miga Motor Company, was used in the next step of experimental research. Its dimensions are shown in Figure 7a. The operating principle of the actuator is based on changing the shape (shortening) of the active element (thin wire made of nitinol, which is a shape memory alloy), which allows the movement of the active part (the metal, longitudinal plate). The actuator is controlled by a pulse width modulation (PWM) signal, which means that the speed and range of displacements depend on the duty cycle, and has a construction limitation—the maximum displacement (stroke) is 1.66 mm. The passive part of the actuator was immobilized during the examination by clamping it between the jaws of the vice (Figure 7a). The preload of the actuator was carried out by suspending weights on a pin, which was connected to the active element of the actuator (Figure 6c). The preload vector direction was parallel to the actuator displacement direction.

#### 3.2.1. Research Program

In the first phase of the research, the relationship between the duty cycle and the maximum extension of the active element of the actuator was analysed for different preloads (achieved by hanging an additional mass of 0 g, 1,040.57 g or 2,037.89 on the active part). The range of the duty cycle was set to 0–6.7% for the case without preload, 0–10% for a load of 10.21 N, and 0–20% for a load of 19.99 N. In each series of the tests, the value of the duty cycle was gradually increased. The duration of a given increment has been adjusted to observe the displacement stabilisation. Three measurement series were carried out for each preload. After each series, the control signal was reset to zero. An example of the displacement diagram obtained for the actuator without preload is presented in Figure 7b. A gradual increase in displacement is visible. After increasing the duty cycle, an asymptotic increase in displacement was noted.

#### 3.2.2. Results

**Figure 8:**
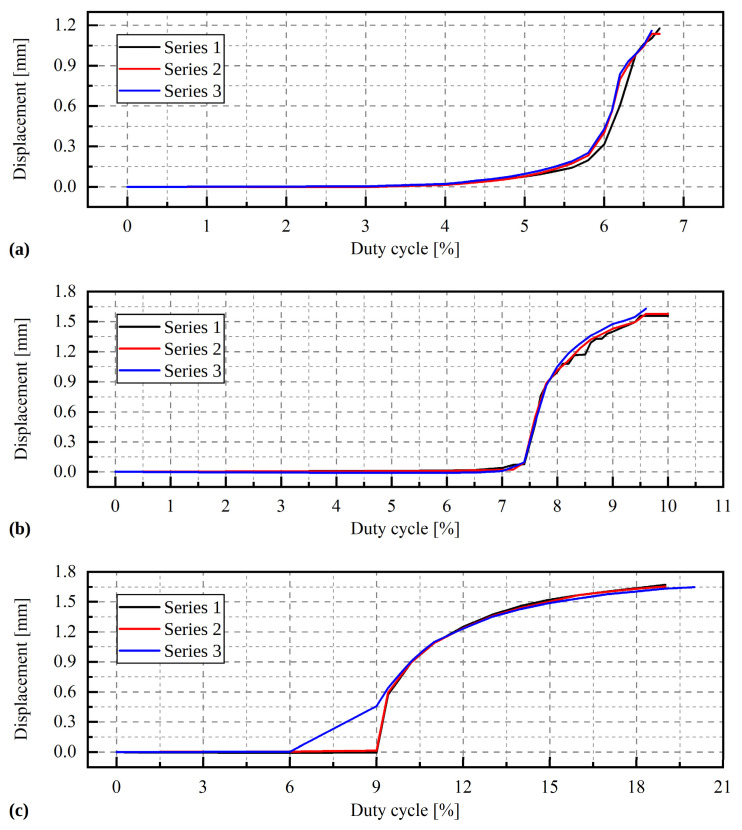
Graphs showing displacement versus time (**a**) for load 0 N; (**b**) for load 9.81 N, and (**c**) for load 19.62 N.

The obtained characteristics of the SMA actuator, i.e., the maximum displacement obtained for a given duty cycle, are presented in Figure 8. The values shown in the graphs have been normalised to a minimum displacement value (using a zero-fill factor). The clear non-linear nature of the relationship between load and displacement is visible for all analysed cases. In the initial period, a notable change in the duty cycle is accompanied by a slight increase in displacements, whereas for higher values of the duty cycle, a higher displacements increase is visible. The extension range of the SMA actuator without preload is much lower than the value declared by the producer; it is about 1.2 mm (72.28% of the declared range). This is due to the one-way action of the actuator. A PWM control signal shortens the nitinol wire and moves the active element of the actuator. Cutting off the signal causes the wire to cool slowly and return to its original state. If during this process any resistance to motion occurs (e.g., friction between the active and passive actuator elements), the actuator is unable to return to the starting position and therefore the range of movement is limited. This is not visible if the actuator is preloaded (the load vector has the same direction as the displacement of the actuator, but its sense is the opposite). The operating range of the preloaded actuator is therefore consistent with the range declared by the producer. The duty cycle of the preloaded actuator is 9–19% at a load of 19.99 N. The increase in the preload causes a shift and extension of the operating range, e.g., for a load of 19.99 N, significant displacements occur for the duty cycle in the range of 9–20%.

### 3.3. Stepper Motor

**Figure 9:**
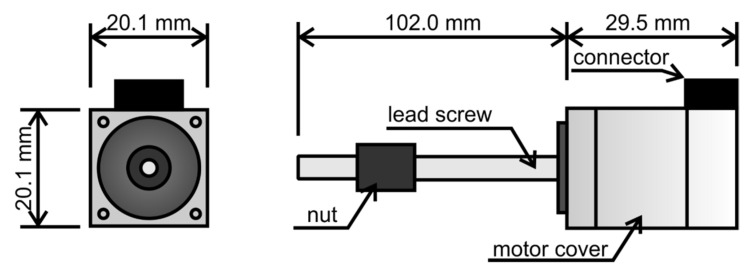
Dimensions of the MLS08A05-M04010S10160N-A000-XT1 stepper motor.

The dimensions of the actuator are shown in Figure 9. The tested element consists of a rectangular cover, which contains a hybrid stepper motor and a threaded rod protruding from it, on which a backlash-free nut is mounted as standard. Therefore, the stepper motor can be used as a rotary or linear actuator. Thanks to the high manufacturing precision, the element ensures stable operation; it allows smooth regulation up to 5800 revolutions (each of which results in a rotation of 1.8°).

Due to the nature of stepper motor operation, the static tests have been omitted. The dynamic analysis was performed to determine the characteristics of the actuator in preload terms. For this purpose, the angle of rotation was measured as a function of time over the entire operating range of the motor (eight hundred revolutions). The additional load was applied using the lift, which was placed on the lead screw nut. Due to the special construction of the nut and the frictional force acting between the elements, the operation of the stepper motor caused only linear movement of the nut, without rotation. The stepper motor has been tested in both directions to analyse the influence of the force whose direction is the same or opposite to the direction of movement. In the following test series, the acceleration of the stepper motor was also modified.

#### Results

**Figure 10:**
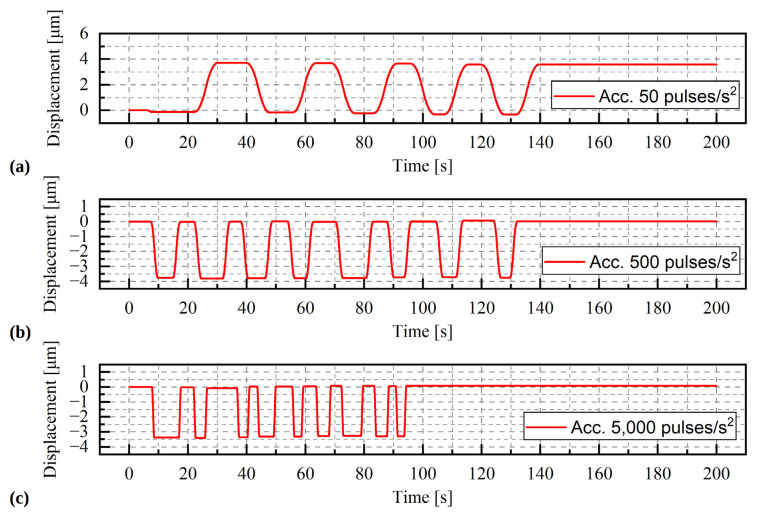
Displacement of the stepper motor with a load of 9.81 N. During the test the acceleration was change (**a**) acceleration = 50 pulses/s^2^, (**b**) acceleration = 500 pulses/s^2^, (**c**) acceleration = 5000 pulses/s^2^.

**Figure 11:**
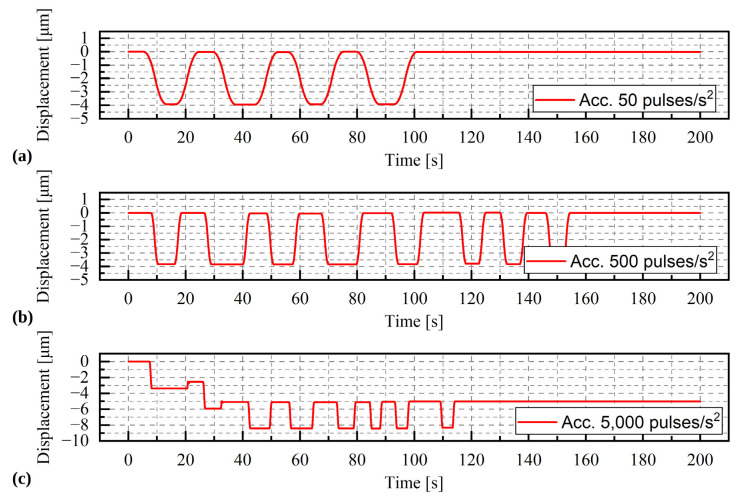
Displacement of the stepper motor with a load of 14.72 kg. During the test the acceleration was change (**a**) acceleration = 50 pulses/s^2^, (**b**) acceleration = 500 pulses/s^2^, (**c**) acceleration = 5000 pulses/s^2^.

**Figure 12:**
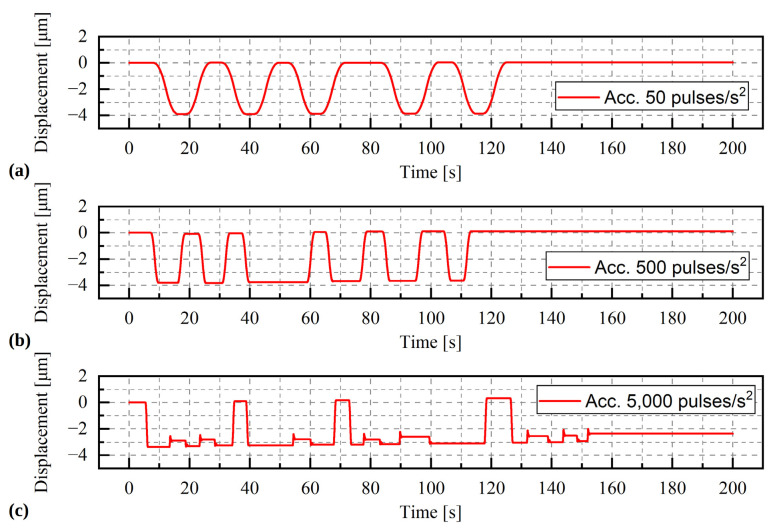
Displacement of the stepper motor with a load of 19.62 kg. During the test the acceleration was change (**a**) acceleration = 50 pulses/s^2^, (**b**) acceleration = 500 pulses/s^2^, (**c**) acceleration = 5000 pulses/s^2^.

The results of the selected series of measurements are shown in Figure 10, Figure 11 and Figure 12. During the tests, only the angle of rotation of the threaded rod was measured. The values of linear displacements, which are presented on the graphs, were obtained by multiplying the measured values of the rotation angle by a constant value of 0.00005/π mm. For a given range of rotation, three phases of movement can be distinguished: the acceleration phase, the phase of movement at a constant speed and the deceleration phase. As the rotational acceleration increases, the proportion of the first or third phase to the second phase decreases. High repeatability of results was observed in all series of measurements at a load of 9.81 N (Figure 10a). The displacement waveforms are almost identical for a given acceleration and it is difficult to notice any influence of the direction of rotation on the load. Whereas for a greater mass, i.e., 1.5 kg and 2 kg (Figure 11 and Figure 12), such a situation occurs only for accelerations of 50 pulses/s^2^ and 500 pulses/s^2^. In the last test, conducted at an acceleration of 5000 pulses/s^2^, the curve is irregular. The additional load was large enough to prevent rotation of the stepper motor.

## 4. Discussion and Conclusions

The process of designing an active foil bearing presents many challenges in terms of foil bearing characteristics. When designing this type of bearing, many important elements must be taken into account in the active control system. From a practical point of view, it is very important to determine the characteristics of the actuators because they will directly affect the characteristics of the active foil bearing. Their reaction time, control accuracy and maximum force they can transmit will determine the limitations of active foil bearings. Based on this article, it is possible not only to assess the use of the tested actuators in similar designs, but also, through the prism of these results, to get a more complete picture of the characteristics of all active foil bearings that are presented in the literature.

This article presents the results of experimental tests conducted on three selected actuators that use various smart materials or stepper motors. Their common feature is the ability to generate a linear displacement or force, but they have different operating ranges and accuracy. The differences in actuator performance can be minimized by the proper design of the active foil bearing. For example, it can be divided into two parts, with a single main actuator (as shown in paper No. [30]). Another solution is to divide the active foil bearing into several parts. In some cases, the researchers also use levers to increase the effective displacement generated by the actuators [17]. Due to the variety of designs available on the market, all actuators discussed in this article can be used directly not only in active foil bearings but also in other types of active bearings.

The piezoelectric actuator allows for a smooth and quick change of displacements within a range of −10–50 µm. A preload of up to 98.1 N does not affect its operating range; slight differences during the displacement are observed only at high frequencies. However, it is linked to the method of implementation of the preload, by adding additional masses. The displacement range of the actuator can be increased using levers or other multiplier mechanisms. Its main drawback lies in its hysteretic properties, visible especially in quasi-static tests. In systems that use a piezoelectric element, it may be necessary to use additional measurement sensors to monitor the displacements.

The range of linear displacements generated by the SMA actuator is much wider than that of the piezoelectric stack—up to 1.6 mm, which is accompanied by a high sensitivity to preload. In the analysed range of preload (0–19.62 N), there are visible differences in the relationship between displacement and duty cycle. The SMA actuator also has a long response time and reaches the maximum displacement at the specified value of the duty cycle, which takes from a few seconds to several minutes. The use of this type of actuator significantly limits the possibility of smooth and rapid control of the mechanical system. The big disadvantage of the actuator is its one-way operation. In addition, the tests carried out at different temperatures show a strong dependence between the ambient temperature and the displacement during the duty cycle. As the ambient temperature decreases, the maximum displacement measured for a given duty cycle value also decreases. This is also accompanied by a slower reaching of set values. The possibility of using the SMA actuator in a bearing may therefore require the development of a precise analytical model, which would take into account environmental parameters; otherwise, the long stabilization time of this element may cause difficulties in obtaining the expected displacements.

The stepper motor has the greatest displacement range and low sensitivity to external loads. Due to the nature of its operation, the regulation is not completely smooth. When the motor performs a certain number of steps (revolutions), each of them translates into a linear displacement of 5 µm of the lead screw nut. However, this does not exclude the possibility of precise control using this actuator. Below a certain load level, the stepper motor does not show any sensitivity to external loads. It is only when this level is exceeded that skips or blockages of the displacements (rotations) of the pin are observed. This load limit depends on the acceleration of the motor, and it decreases as the acceleration increases. The stepper motor provides high repeatability even at high operating speeds. Due to the displacement range and the low sensitivity to preload, the use of this motor in active bearings is very promising.

## 5. Patents

Breńkacz, Ł.; Bagiński, P., Patent: Układ oraz sposób sterowania aktywnym łożyskiem foliowym [The arrangement and method of controlling the active foil bearing], Date of Issue: 8 April 2022.
